# Pulmonary neuroendocrine tumors: study of 266 cases focusing on clinicopathological characteristics, immunophenotype, and prognosis

**DOI:** 10.1007/s00432-022-03970-x

**Published:** 2022-03-06

**Authors:** Shuwen Zhang, Jingjing Chen, Rui Zhang, Liqin Xu, Yan Wang, Zaixin Yuan, Xiaohui Hou, Jian Feng

**Affiliations:** 1 Department of Respiratory and Critical Care Medicine, Affiliated Hospital of Nantong University, 20 Xi-Si Road, Nantong, 226001 Jiangsu China; 2 Department of Pathology, Affiliated Hospital of Nantong University, Nantong, Jiangsu China; 3 Nantong University, Nantong, Jiangsu China

**Keywords:** Clinicopathological characteristics, Platelet–lymphocyte ratio, Lymphocyte–monocyte ratio, Carcinoembryonic antigen, Overall survival, Pulmonary neuroendocrine tumor

## Abstract

**Objective:**

Pulmonary neuroendocrine tumors (PNETs) consist of small-cell lung cancer (SCLC), large-cell neuroendocrine carcinoma (LCNEC), typical carcinoid (TC), and atypical carcinoid (AC). We aimed to analyze the immunophenotypic, metastatic, and prognostic risk factors for PNETs.

**Materials and methods:**

A total of 266 patients with PNETs were enrolled, including 219 patients with SCLC, 18 patients with LCNEC, 11 patients with TC, and 18 patients with AC. Clinicopathological characteristics and immunophenotypes were compared among the subtypes of PNETs. Risk factors for metastasis, progression-free survival (PFS), and overall survival (OS) were analyzed.

**Results:**

Thyroid transcription factor-1 (TTF-1) and the Ki-67 index were significantly different among subtypes of PNETs (all *P* < 0.05). Smoking (OR, 2.633; *P* = 0.031), high pretreatment carcinoembryonic antigen (CEA > 5 ng/ml: OR, 3.084; *P* = 0.014), and poorly differentiated pathotypes (*P* = 0.001) were independent risk factors for lymph-node metastasis. Smoking (OR, 2.071; *P* = 0.027) and high pretreatment CEA (OR, 2.260; *P* = 0.007) were independent risk factors for distant metastasis. Results of the multivariate Cox regression model showed pretreatment CEA (HR, 1.674; *P* = 0.008) and lymphocyte–monocyte ratio (LMR) (HR = 0.478, *P* = 0.007) were significantly associated with PFS; BMI (*P* = 0.031), lymph-node metastasis (HR = 4.534, *P* = 0.001), poorly differentiated pathotypes (*P* = 0.015), platelet–lymphocyte ratio (PLR) (HR = 2.305, *P* = 0.004), and LMR (HR = 0.524, *P* = 0.045) were significantly associated with OS.

**Conclusions:**

PNETs are a group of highly heterogeneous tumors with different clinical manifestations, pathological features, and prognoses. Knowing clinicopathological characteristics and immunophenotypes of PNETs is significant for diagnosis. Pretreatment PLR, LMR, and CEA have certain value in the prognosis of PNETs.

## Introduction

Pulmonary neuroendocrine tumors (PNETs) are a unique subtype of primary lung cancer. It has been reported that PNETs represented 20% of all lung cancers (Gustafsson et al. 2008). According to the 2015 World Health Organization (WHO) classification of lung tumors, PNETs include small-cell lung cancer (SCLC), large-cell neuroendocrine carcinoma (LCNEC), typical carcinoid (TC), and atypical carcinoid (AC). TC and AC are well-differentiated PNETs, whereas LCNEC and SCLC are poorly differentiated PNETs (Travis et al. 2015).

Even though PNET is rare, the incidence and prevalence are increasing, and the prognosis (especially SCLC and LCNEC) is terrible (Dasari et al. 2017). Fortunately, people are currently paying more attention to this kind of disease, especially for epidemiology, immunohistochemical molecular characteristics, and diagnosis. Current diagnostic methods primarily depend on neuroendocrine morphology and immunohistochemistry, such as cellular morphology, nuclear divisions, and the Ki-67 index. The 2015 WHO classification of lung tumors suggested that synaptophysin (Syn), chromogranin A (CgA), and CD56 are recommended as neuroendocrine (NE) markers, and Syn and CgA were suggested as the first-hand choice (Travis et al. 2015). Rekhtman (2010) revealed a mean (range) Ki-67 labeling index of 1.5 (0–2.3%) for typical carcinoid tumors, 7.7 (0–17%) for atypical carcinoid tumors, and 64 (25–96%) for SCLC. Marchevsky et al. (2018) pointed out that the best cut-off value was Ki-67 < 5%, which had great significance for diagnosis and prognosis.

Current strategies for tumor treatment include chemotherapy, radiotherapy, immunotherapy, targeted therapy, and surgery. The therapeutic options vary in the subtypes of PNETs. SCLC is initially highly sensitive to chemotherapy and radiotherapy, but most patients usually relapse and acquire resistant disease. The indications and results of surgical resection for SCLC remain controversial, and only a minority of patients with SCLC qualify for surgical resection. Programmed death 1 (PD-1) and programmed death ligand 1 (PD-L1) immune checkpoint inhibitors show good clinical activity in SCLC treatment. Atezolizumab combined with etoposide/carboplatin is recommended as the first-line treatment of extensive SCLC (Dingemans et al. 2021). However, there is no effective targeted therapy for SCLC. The first-line treatment modalities of LCNEC are quite different. In early stages, surgical resection is the preferred treatment, and patients can benefit from adjuvant chemotherapy at all operable stages (IA–IIIA). In its advanced stages, experience from the Dutch PALGA network has suggested that platinum-gemcitabine/taxanes may perform better than traditional platinum–etoposide approaches (Derks et al. 2017, 2018). For lung carcinoids (LCs), surgery is the preferred treatment. Large retrospective studies have reported no benefit of adjuvant therapy in either TCs or ACs. Therefore, the authors do not recommend routine adjuvant therapy in LCs (Anderson Jr et al. 2017; Daddi et al. 2014; García-Yuste et al. 2007; Nussbaum et al. 2015; Steuer et al. 2015).

To analyze the prognosis of PNETs, most previous studies have focused on Western populations. These large population-based studies usually download cases of PNETs in some databases for analyses [such as Surveillance, Epidemiology, and End Results (SEER) database] (Dasari et al. 2017; Doll et al. 2018). Databases provide clinical diagnosis, treatment, and prognosis information of different histopathological cancers and can help us better understand the disease. However, even the same disease may have diverse characteristics in different countries and regions. Their sample size is large, but practical applicability is limited. There are also some studies collecting cases in real clinical practice, but the sample size is small (Kim et al. 2020; Yeh and Chou 2014). Prior studies have implied that gender, age at diagnosis, tumor diameter, metastasis, stage, and first-line treatment modalities are associated with the prognosis of PNETs (Yang et al. 2019; Yeh and Chou 2014). However, only a limited number of studies have analyzed the clinical detection and survival outcomes of lung neuroendocrine neoplasms. Yet, high pretreatment carcinoembryonic antigen (CEA), neutrophil–lymphocyte ratio (NLR), platelet–lymphocyte ratio (PLR), and low lymphocyte–monocyte ratio (LMR) are tightly associated with poor prognosis of non-small-cell lung cancer (NSCLC) (Chen et al. 2018; Grunnet and Sorensen 2012; Kuo et al. 2020). Thus, large-scaled studies based on Chinese population are urgently needed to explore the relationship between clinical tests and the prognosis of pulmonary neuroendocrine tumors.

In our study, we involved 266 lung neuroendocrine neoplasms patients who had received treatments at our institution over the last 8 years and analyzed the clinicopathological characteristics and immunohistochemical profile. Simultaneously, clinical test indices were included to generalize metastatic risk factors for PNETs and to identify the influence factors of progression-free survival (PFS) and overall survival (OS).

## Materials and methods

### Materials

We collected patients’ clinical and pathological data from patients who were diagnosed with PNETs and given treatments at the Affiliated Hospital of Nantong University from January 2012 to December 2020. PNETs were diagnosed based on the 2015 WHO diagnostic criteria. These patients’ chest imaging examination and pretreatment blood examination results are available. All of them received at least one treatment at our institution. The exclusion criteria included the following: combined with other primary tumors and/or acute infectious diseases and failure to follow-up.

### Methods

The participants’ general clinical and anthropometric information were obtained from medical records and recorded at baseline, such as name, age, gender, body mass index (BMI), smoking history, laterality, and tumor diameter. Cases were divided into SCLC, LCNEC, TC, and AC according to the grading and classification criteria of pulmonary neuroendocrine tumors of the 2015 WHO. For the stage of PNETs, the two-stage system was used, in which limited-stage PNETs are defined as disease confined to a single, tolerable radiation port (stage I–III), and extensive-stage PNETs are the diseases that has extended beyond a single tolerable port (stage IV). We retrospectively analyzed tumors diagnosis, stages, lymph-node metastasis, distant metastasis, immunohistochemical molecular characteristics, pretreatment CEA, NLR, PLR, LMR, and therapeutic measures. Syn, CgA, Cytokeratin 18 (CK18), TTF-1, Napsin A, Cytokeratin 5/6 (CK5/6), p63, and Ki-67 index were analyzed as a representative molecule for immunohistochemistry. Pretreatment blood sampling was performed to measure the neutrophil, lymphocyte, monocyte, platelet, and CEA levels. The NLR was determined as the neutrophil count divided by the lymphocyte count, PLR was determined as the platelet count divided by the lymphocyte count, and LMR was defined as the absolute lymphocyte count/the absolute monocyte count. PFS was calculated from the date of treatment initiation to the date of progression or death or last follow-up, and OS was defined as the time from the date of treatment initiation to the date of death or last follow-up. Follow-up was terminated on December 31, 2021. The above clinicopathological characteristics were grouped by whether there was metastasis, disease progression, and death separately, implying risk factors for metastasis and survival.

### Statistical analysis

Clinicopathological characteristics and immunophenotype of SCLC, LCNEC, TC, and AC were compared using the Pearson’s chi-squared test and Kruskal–Wallis *H* test. Frequencies and percentages are reported for categorical variables, and medians with range or interquartile range are reported for continuous variables. Receiver-operating characteristic (ROC) curve analysis was performed to analyze the area under the ROC curve (AUC), and the Youden Index was used to identify the optimal cut-off values for NLR, PLR, and LMR. Univariate and multivariate logistic regression models were applied to identify risk factors for metastasis, and accurate estimate of odd ratios (ORs) and 95% confidence intervals (95% CIs) were reported. To analyze the prognostic factors, univariate and multivariate Cox proportional hazard models were used, and the hazard ratios (HRs) and 95% CIs were reported. Survival analysis was conducted and the significance of differences among groups was tested using the log-rank test. PFS and 5-year OS were investigated using the Kaplan–Meier method. Above analyses were performed using SPSS statistical package version 26.0 (SPSS, Chicago, IL, USA). *P* value < 0.05 was considered statistically significant. GraphPad Prism7 software (GraphPad Software, San Diego, CA, USA) was used to generate the ROC curves and survival curves.

## Results

### The optimal cut-off point of PLR, NLR, and LMR

**Fig. 1:**
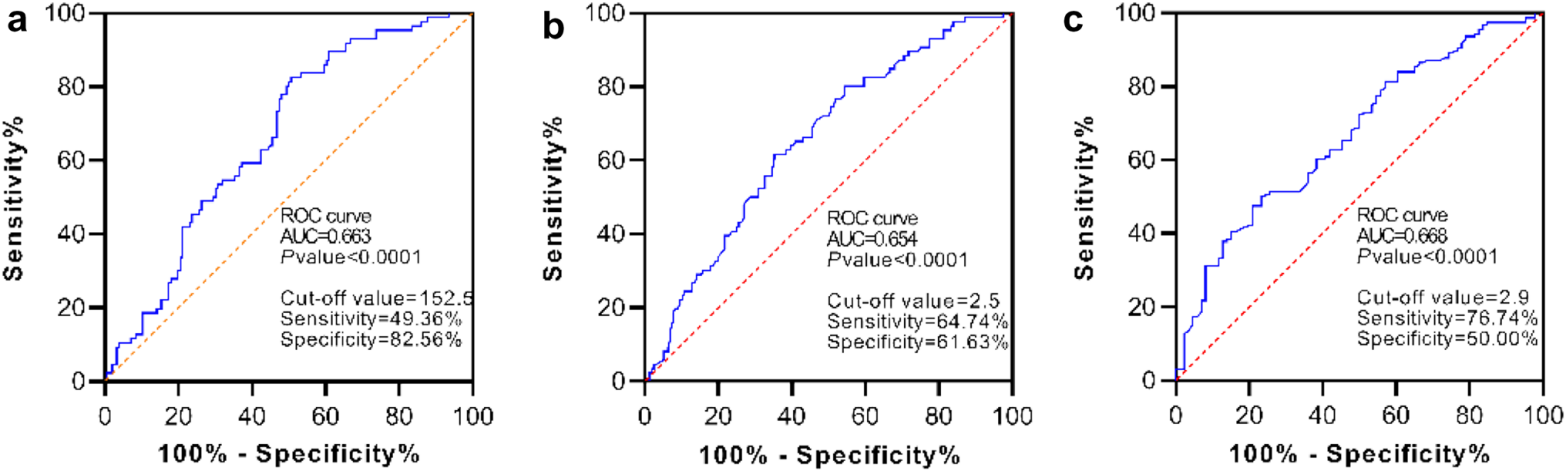
ROC curve for immune-inflammation indices. **a** ROC curve for PLR; **b** ROC curve for NLR; **c** ROC curve for LMR. Abbreviations: *ROC curve* receiver-operating characteristic curve; *PLR* platelet–lymphocyte ratio; *NLR* neutrophil–lymphocyte ratio; *LMR* lymphocyte–monocyte ratio

To determine the potential prognostic role of NLR, PLR, and LMR in PNETs, ROC analysis was performed to identify the optimal cut-off point of these immune-inflammation indices. ROC curve analysis indicated an optimal cut-off PLR of 152.5 (AUC = 0.663, 95%CI = 0.594–0.731, sensitivity = 49.36%, specificity = 82.56%), an optimal cut-off NLR of 2.5 (AUC = 0.654, 95%CI = 0.584–0.725, sensitivity = 64.74%, specificity = 61.63%), and optimal cut-off LMR of 2.9 (AUC of 0.668, 95%CI = 0.598–0.738, sensitivity = 76.74%, specificity = 50.00%) (Fig. 1). For each immune-inflammation index, patients were divided into two groups for further analysis [PLR ≤ 152.5 (low) and PLR > 152.5 (high); NLR ≤ 2.5 (low) and NLR > 2.5 (high); LMR ≤ 2.9 (low) and LMR > 2.9 (high)].

### Clinical characteristics’ comparison among PNETs

A total of 266 patients with PNETs were enrolled in this study, including 219 (82.32%) SCLC patients, 18 (6.77%) LCNEC patients, 11 (4.14%) and 18 (6.77%) patients of TC and AC, respectively. Clinicopathological characteristics according to PNET subtype are summarized in Table 1. Gender, age, lymph-node metastasis, distant metastasis, stage, NLR, first-line treatment modalities, PFS, and OS showed significant differences among SCLC, LCNEC, AC, and TC cases (all *P* < 0.05). PNETs were commonly seen in males (86.09%). Compared with LCNEC, AC, and TC patients, the proportion of males was much higher in SCLC cases (89.95% vs. 77.78% vs. 54.55% vs. 66.67%; *P* = 0.001). For the population included in this study, SCLC tended to occur among elderly individuals (≥ 60 years: 75.80% vs. 61.11% vs. 45.45% vs. 55.56%; *P* = 0.030), most frequently with metastasis (lymph-node metastasis: 89.04% vs. 72.22% vs. 36.36% vs. 38.89%, *P* = 0.000; distant metastasis: 43.84% vs. 22.22% vs. 0.00% vs. 11.11%, *P* = 0.000) and in an advanced stage at the time of diagnosis (extensive stage/stage IV: 46.58% vs. 27.78% vs. 18.18% vs. 16.67%, *P* = 0.012). The pretreatment NLR was higher in SCLC and LCNEC (> 2.5: 63.51% vs. 64.71% vs. 18.18% vs. 27.78%, *P* = 0.001), but pretreatment PLR, LMR and CEA did not reveal significant differences in the intergroup comparison at baseline. First-line treatment modalities (*P* = 0.000), median progression-free survival (mPFS: 6.62 months vs. 11.83 months vs. 52.86 months vs. 17.39 months, *P* = 0.000), and median overall survival (mOS: 14.97 months vs. 21.42 months vs. 54.43 months vs. 32.02 months, *P* = 0.000) showed significantly differences among the groups.

**Table 1:** Clinical characteristics’ comparison among PNETs

	All	SCLC	LCNEC	TC	AC	*χ*^2^	*P* value
Gender						16.157	0.001*
Male	229 (86.09)	197 (89.95)	14 (77.78)	6 (54.55)	12 (66.67)		
Female	37 (13.91)	22 (10.05)	4 (22.22)	5 (45.45)	6 (33.33)		
Age at diagnosis (years)						8.917	0.030*
< 60	74 (27.82)	53 (24.20)	7 (38.89)	6 (54.55)	8 (44.44)		
≥ 60	192 (72.18)	166 (75.80)	11 (61.11)	5 (45.45)	10 (55.56)		
BMI (kg/m^2^)						1.963	0.925
< 18.5	15 (5.64)	14 (6.39)	0 (0.00)	0 (0.00)	1 (5.56)		
18.5–23.9	139 (52.26)	115 (52.51)	9 (50.00)	7 (63.64)	8 (44.44)		
> 24	112 (42.11)	90 (41.10)	9 (50.00)	4 (36.36)	9 (50.00)		
Smoking						6.341	0.096
No	118 (44.36)	92 (42.01)	7 (38.89)	8 (72.73)	11 (61.11)		
Yes	148 (55.64)	127 (57.99)	11 (61.11)	3 (27.27)	7 (38.89)		
Tumor size (cm)						15.069	0.058
≤ 3	100 (37.59)	74 (33.79)	7 (38.89)	8 (72.73)	11 (61.11)		
3–5	88 (33.08)	74 (33.79)	9 (50.00)	1 (9.09)	4 (22.22)		
5–7	49 (18.42)	44 (20.09)	2 (11.11)	2 (18.18)	1 (5.56)		
> 7	29 (10.90)	27 (12.33)	0 (0.00)	0 (0.00)	2 (11.11)		
Laterality						5.437	0.410
Left	107 (40.23)	92 (42.01)	7 (38.89)	5 (45.45)	3 (16.67)		
Right	148 (55.64)	117 (53.42)	11 (61.11)	6 (54.55)	14 (77.78)		
Others	11 (4.14)	10 (4.57)	0 (0.00)	0 (0.00)	1 (5.56)		
Lymph-node metastasis						37.945	0.000*
No	47 (17.67)	24 (10.96)	5 (27.78)	7 (63.64)	11 (61.11)		
Yes	219 (82.33)	195 (89.04)	13 (72.22)	4 (36.36)	7 (38.89)		
Distant metastasis						18.470	0.000*
No	164 (61.65)	123 (56.16)	14 (77.78)	11 (100.00)	16 (88.89)		
Yes	102 (38.35)	96 (43.84)	4 (22.22)	0 (0.00)	2 (11.11)		
Stage						10.553	0.012*
Limited stage/stage I–III	154 (57.89)	117 (53.42)	13 (72.22)	9 (81.82)	15 (83.33)		
Extensive stage/stage IV	112 (42.11)	102 (46.58)	5 (27.78)	2 (18.18)	3 (16.67)		

	All	SCLC	LCNEC	TC	AC	*χ*^2^	*P* value
CEA (ng/ml)						7.101	0.069
≤ 5	151 (56.77)	117 (54.42)	12 (66.67)	10 (90.91)	12 (66.67)		
> 5	111 (41.73)	98 (45.58)	6 (33.33)	1 (9.09)	6 (33.33)		
PLR						6.988	0.066
≤ 152.5	157 (61.09)	121 (57.35)	14 (82.35)	8 (72.73)	14 (77.78)		
> 152.5	100 (38.91)	90 (42.65)	3 (17.65)	3 (27.27)	4 (22.22)		
NLR						16.847	0.001*
≤ 2.5	105 (40.86)	77 (36.49)	6 (35.29)	9 (81.82)	13 (72.22)		
> 2.5	152 (59.14)	134 (63.51)	11 (64.71)	2 (18.18)	5 (27.78)		
LMR						5.576	0.130
≤ 2.9	104 (40.47)	92 (43.60)	6 (35.29)	2 (18.18)	4 (22.22)		
> 2.9	153 (59.53)	119 (56.40)	11 (64.71)	9 (81.82)	14 (77.78)		
Treatment strategy						78.119	0.000*
Chemotherapy	179 (67.29)	167 (76.26)	6 (33.33)	1 (9.09)	5 (27.78)		
Chemotherapy + radiotherapy	5 (1.88)	5 (2.28)	0 (0.00)	0 (0.00)	0 (0.00)		
Surgery	27 (10.15)	6 (2.74)	5 (27.78)	7 (63.64)	9 (50.00)		
Surgery + chemotherapy/radiotherapy	40 (15.04)	29 (13.24)	6 (33.33)	1 (9.09)	4 (22.22)		
Others	15 (5.64)	12 (5.48)	1 (5.56)	2 (18.18)	0 (0.00)		
PFS	6.93 (3.77,13.45)	6.62 (3.56,10.42)	11.83 (2.32,35.09)	52.86 (26.30,54.70)	17.39 (5.74,41.93)		0.000*
OS	16.53 (9.61,30.1)	14.97 (9.37,26.48)	21.42 (4.98,44.40)	54.43 (32.70,82.00)	32.02 (17.58,41.93)		0.000*

*PNETs* pulmonary neuroendocrine tumors; *SCLC* small-cell lung cancer; *LCNEC* large-cell neuroendocrine carcinoma; *AC* atypical carcinoid; TC, typical carcinoid; *BMI* body mass index; *CEA* carcinoembryonic antigen; *PLR* platelet–lymphocyte ratio; *NLR* neutrophil–lymphocyte ratio; *LMR* lymphocyte–monocyte ratio; *PFS* progression-free survival; *OS* overall survival

*P < 0.05

### Immunohistochemical molecular characteristics’ comparison among PNETs

To compare the differences in immunohistochemical profiles among PNETs, Syn, CgA, CK18, TTF-1, Napsin A, CK5/6, Ki-67, and p63 were examined (Table 2). SCLC had a higher positive rate of TTF-1 (89.50% vs. 52.94% vs. 80.00% vs. 72.22%, *P* = 0.000). The Ki-67 index was significantly higher in SCLC and LCNEC than in carcinoid tumors (the median: 80.00% vs. 75.00% vs. 2.00% vs 30.00%, *P* = 0.000). Other immunohistochemical molecules (such as Syn, CgA, CK18, Napsin A, CK5/6, p63) did not show significant differences among the subtypes of PNETs.

**Table 2:** Immunohistochemical molecular characteristics comparison among PNETs

	All	SCLC	LCNEC	TC	AC	*χ*^2^	*P* value
Syn						6.767	0.052
Negative	26 (10.32)	18 (8.74)	5 (27.78)	2 (18.18)	1 (5.88)		
Positive	226 (89.68)	188 (91.26)	13 (72.22)	9 (81.82)	16 (94.12)		
CgA						5.875	0.112
Negative	76 (31.67)	63 (32.14)	9 (50.00)	2 (20.00)	2 (12.50)		
Positive	164 (68.33)	133 (67.86)	9 (50.00)	8 (80.00)	14 (87.50)		
CK18						4.313	0.210
Negative	6 (2.93)	4 (2.35)	1 (6.25)	1 (12.50)	0 (0.00)		
Positive	199 (97.07)	166 (97.65)	15 (93.75)	7 (87.50)	11 (100.00)		
TTF-1						16.618	0.000*
Negative	36 (14.69)	21 (10.50)	8 (47.06)	2 (20.00)	5 (27.78)		
Positive	209 (85.31)	179 (89.50)	9 (52.94)	8 (80.00)	13 (72.22)		
Napsin A						4.153	0.183
Negative	221 (97.36)	177 (97.79)	16 (88.89)	11 (100.00)	17 (100.00)		
Positive	6 (2.64)	4 (2.21)	2 (11.11)	0 (0.00)	0 (0.00)		
CK5/6						2.247	0.466
Negative	193 (95.54)	151 (95.57)	16 (88.89)	9 (100.00)	17 (100.00)		
Positive	9 (4.46)	7 (4.43)	2 (11.11)	0 (0.00)	0 (0.00)		
Ki67 (%)	80.00 (60.00,80.00)	80.00 (70.00,86.25)	75.00 (52.50,80.00)	2.00 (1.00,2.75)	30.00 (20.00,50.00)		0.000*
P63						3.041	0.339
Negative	135 (86.54)	103 (83.74)	11 (91.67)	8 (100.00)	13 (100.00)		
Positive	21 (13.46)	20 (16.26)	1 (8.33)	0 (0.00)	0 (0.00)		

*PNETs* pulmonary neuroendocrine tumors; *SCLC* small-cell lung cancer; *LCNEC* large-cell neuroendocrine carcinoma; *AC* atypical carcinoid; *TC* typical carcinoid; *Syn* synaptophysin; *CgA* chromogranin A; *CK18* Cytokeratin 18; *TTF-1* thyroid transcription factor-1; *CK5/6* Cytokeratin 5/6

^*^P < 0.05

### Metastatic risk factors’ analysis of PNETs

We employed univariate logistic regression analysis to explore the risk factors for metastasis. As shown in Table 3, gender (lymph-node metastasis: female, OR 0.322, 95% CI 0.149–0.693, *P* = 0.004; distant metastasis: female, OR 0.396, 95% CI 0.173–0.905, *P* = 0.028), age at diagnosis (lymph-node metastasis: ≥ 60 years, OR 2.023, 95% CI 1.048–3.906, *P* = 0.036; distant metastasis: ≥ 60 years, OR 2.204, 95% CI 1.216–3.995, *P* = 0.009), smoking (lymph-node metastasis: OR 2.627, 95% CI 1.367–5.050, *P* = 0.004; distant metastasis: OR 1.962, 95% CI 1.177–3.269, *P* = 0.010), tumor size (taking tumor size < 3 cm as reference, lymph-node metastasis: 3–5 cm, OR 3.000, 95% CI 1.399–6.434, *P* = 0.005, 5–7 cm, OR 10.071, 95% CI 2.297–44.168, *P* = 0.002. Distant metastasis: 3–5 cm, OR 1.880, 95% CI 1.013–3.487, *P* = 0.045, 5–7 cm, OR 2.965, 95% CI 1.448–6.070, *P* = 0.003; > 7 cm, OR 3.503, 95% CI 1.486–8.257, *P* = 0.004), and pathotypes (taking SCLC as reference, lymph-node metastasis: LCNEC, OR 0.320, 95% CI 0.105–0.976, *P* = 0.045; TC, OR 0.070, 95% CI 0.019–0.258, *P* = 0.000; AC, OR 0.078, 95% CI 0.028–0.221, *P* = 0.000. Distant metastasis: AC, OR 0.160, 95% CI 0.036–0.713, *P* = 0.016), pretreatment CEA (lymph-node metastasis: > 5 ng/ml, OR 3.678, 95% CI 1.693–7.991, *P* = 0.001; distant metastasis: > 5 ng/ml, OR 2.557, 95% CI 1.531–4.272, *P* = 0.000), PLR (lymph-node metastasis: > 152.5, OR 2.407, 95% CI 1.162–4.988, *P* = 0.018; distant metastasis: > 152.5, OR: 2.249, 95% CI 1.337–3.782, *P* = 0.002), NLR (lymph-node metastasis: > 2.5, OR 2.053, 95% CI 1.083–3.891, *P* = 0.027; distant metastasis: > 2.5, OR 2.402, 95% CI 1.397–4.128, *P* = 0.002), LMR (lymph-node metastasis: > 2.9, OR 0.334, 95% CI 0.158–0.706, *P* = 0.004; distant metastasis: > 2.9, OR 0.350, 95% CI 0.208–0.591, *P* = 0.000) were significantly associated with metastasis. We further conducted multivariate logistic regression analysis to explore the independent risk factors for metastasis. Smoking (OR 2.633; 95% CI 1.093–6.345; *P* = 0.031), pathotypes (taking SCLC as reference, TC, OR 0.139, 95% CI 0.028–0.688, *P* = 0.016; AC, OR 0.105, 95% CI 0.031–0.353, *P* = 0.000), and pretreatment CEA (> 5 ng/ml, OR 3.084; 95% CI 1.256–7.572; *P* = 0.014) were proved to be the independent factors of lymph-node metastasis. Smoking (OR 2.071; 95% CI 1.084–3.956; *P* = 0.027) and pretreatment CEA (> 5 ng/ml, OR: 2.260; 95% CI 1.252–4.080; *P* = 0.007) were the independent risk factors for distant metastasis (Table 3).

**Table 3:** Analysis of potential risk factors for metastasis using univariate and multivariate logistic regression

Risk factors	Lymph-node metastasis				Distant metastasis			
	Univariate logistic regression		Multivariate logistic regression		Univariate logistic regression		Multivariate logistic regression	
	OR (95% CI)	*P* value	OR (95% CI)	*P* value	OR (95% CI)	*P* value	OR (95% CI)	*P* value
Gender								
Male	Reference	–	Reference	–	Reference	–	Reference	–
Female	0.322 (0.149–0.693)	0.004*	1.274 (0.433–3.746)	0.660	0.396 (0.173–0.905)	0.028*	1.538 (0.539–4.386)	0.421
Age at diagnosis (years)								
< 60	Reference	–	Reference	–	Reference	–	Reference	–
≥ 60	2.023 (1.048–3.906)	0.036*	1.280 (0.550–2.976)	0.567	2.204 (1.216–3.995)	0.009*	1.809 (0.901–3.633)	0.095
BMI (kg/m^2^)								
< 18.5	Reference	–			Reference	–		
18.5–23.9	0.380 (0.047–3.038)	0.362			0.665 (0.228–1.934)	0.454		
> 24	0.262 (0.033–2.093)	0.206			0.381 (0.128–1.136)	0.083		
Smoking								
No	Reference	–	Reference	–	Reference	–	Reference	–
Yes	2.627 (1.367–5.050)	0.004*	2.633 (1.093–6.345)	0.031*	1.962 (1.177–3.269)	0.010*	2.071 (1.084–3.956)	0.027*
Tumor size (cm)								
≤ 3	Reference	–	Reference	–	Reference	–	Reference	–
3–5	3.000 (1.399–6.434)	0.005*	2.141 (0.862–5.320)	0.101	1.880 (1.013–3.487)	0.045*	1.409 (0.688–2.885)	0.349
5–7	10.071 (2.297–44.168)	0.002*	6.350 (1.291–31.235)	0.023*	2.965 (1.448–6.070)	0.003*	2.222 (0.982–5.027)	0.055
> 7	2.679 (0.858–8.366)	0.090	1.202 (0.329–4.398)	0.781	3.503 (1.486–8.257)	0.004*	2.232 (0.850–5.860)	0.103
Laterality								
Left	Reference	–			Reference	–		
Right	0.906 (0.470–1.747)	0.769			1.020 (0.610–1.704)	0.941		
Others	0.910 (0.181–4.570)	0.909			2.010 (0.576–7.014)	0.274		
Pathotypes								
SCLC	Reference	–	Reference	–	Reference	–	Reference	–
LCNEC	0.320 (0.105–0.976)	0.045*	0.368 (0.106–1.274)	0.115	0.366 (0.117–1.148)	0.085	0.385 (0.096–1.550)	0.179
TC	0.070 (0.019–0.258)	0.000*	0.139 (0.028–0.688)	0.016*	0.000 (0.000-)	0.999	0.000 (0.000-)	0.999
AC	0.078 (0.028–0.221)	0.000*	0.105 (0.031–0.353)	0.000*	0.160 (0.036–0.713)	0.016*	0.231 (0.045–1.182)	0.078
CEA (ng/ml)								
≤ 5	Reference	–	Reference	–	Reference	–	Reference	–
> 5	3.678 (1.693–7.991)	0.001*	3.084 (1.256–7.572)	0.014*	2.557 (1.531–4.272)	0.000*	2.260 (1.252–4.080)	0.007*
PLR								
≤ 152.5	Reference	–	Reference	–	Reference	–	Reference	–
> 152.5	2.407 (1.162–4.988)	0.018*	1.937 (0.702–5.345)	0.202	2.249 (1.337–3.782)	0.002*	1.404 (0.706–2.792)	0.334
NLR								
≤ 2.5	Reference	–	Reference	–	Reference	–	Reference	–
> 2.5	2.053 (1.083–3.891)	0.027*	0.792 (0.314–1.996)	0.620	2.402 (1.397–4.128)	0.002*	1.351 (0.667–2.738)	0.403
LMR								
≤ 2.9	Reference	–	Reference	–	Reference	–	Reference	–
> 2.9	0.334 (0.158–0.706)	0.004*	0.602 (0.211–1.719)	0.343	0.350 (0.208–0.591)	0.000*	0.567 (0.278–1.159)	0.120

*OR* odd ratio; *95% CI* 95% confidence interval; *BMI* body mass index; *SCLC* small-cell lung cancer; *LCNEC* large-cell neuroendocrine carcinoma; *AC* atypical carcinoid; *TC* typical carcinoid; *CEA* carcinoembryonic antigen; *PLR* platelet–lymphocyte ratio; *NLR* neutrophil–lymphocyte ratio; *LMR* lymphocyte–monocyte ratio

*P < 0.05

### Survival risk factors’ analysis of PNETs

**Fig. 2:**
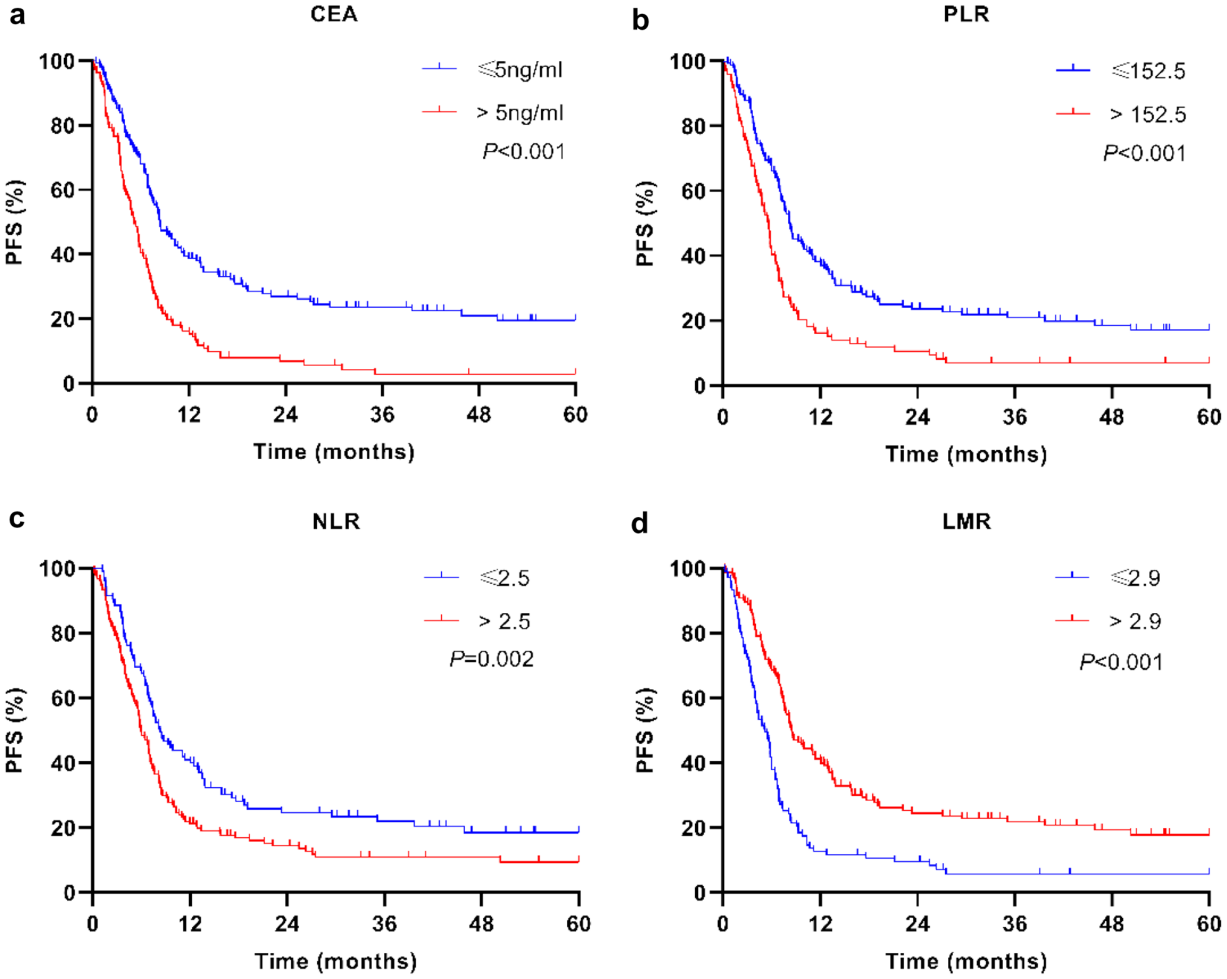
PFS for the PNET patients in matched groups using Kaplan–Meier analysis and log-rank test. **a** PFS among PNET patients based on low pretreatment CEA (≤ 5 ng/ml) and high pretreatment CEA (> 5 ng/ml). **b** PFS among PNET patients based on low pretreatment PLR (≤ 152.5) and high pretreatment PLR (> 152.5). **c** PFS among PNET patients based on low pretreatment NLR (≤ 2.5) and high pretreatment NLR (> 2.5). **d** PFS among PNET patients based on low pretreatment LMR (≤ 2.9) and high pretreatment LMR (> 2.9). Abbreviations: *PFS* progression-free survival; *PNET* pulmonary neuroendocrine tumor; *CEA* carcinoembryonic antigen; *PLR* platelet–lymphocyte ratio; *NLR* neutrophil–lymphocyte ratio; *LMR* lymphocyte–monocyte ratio

**Fig. 3:**
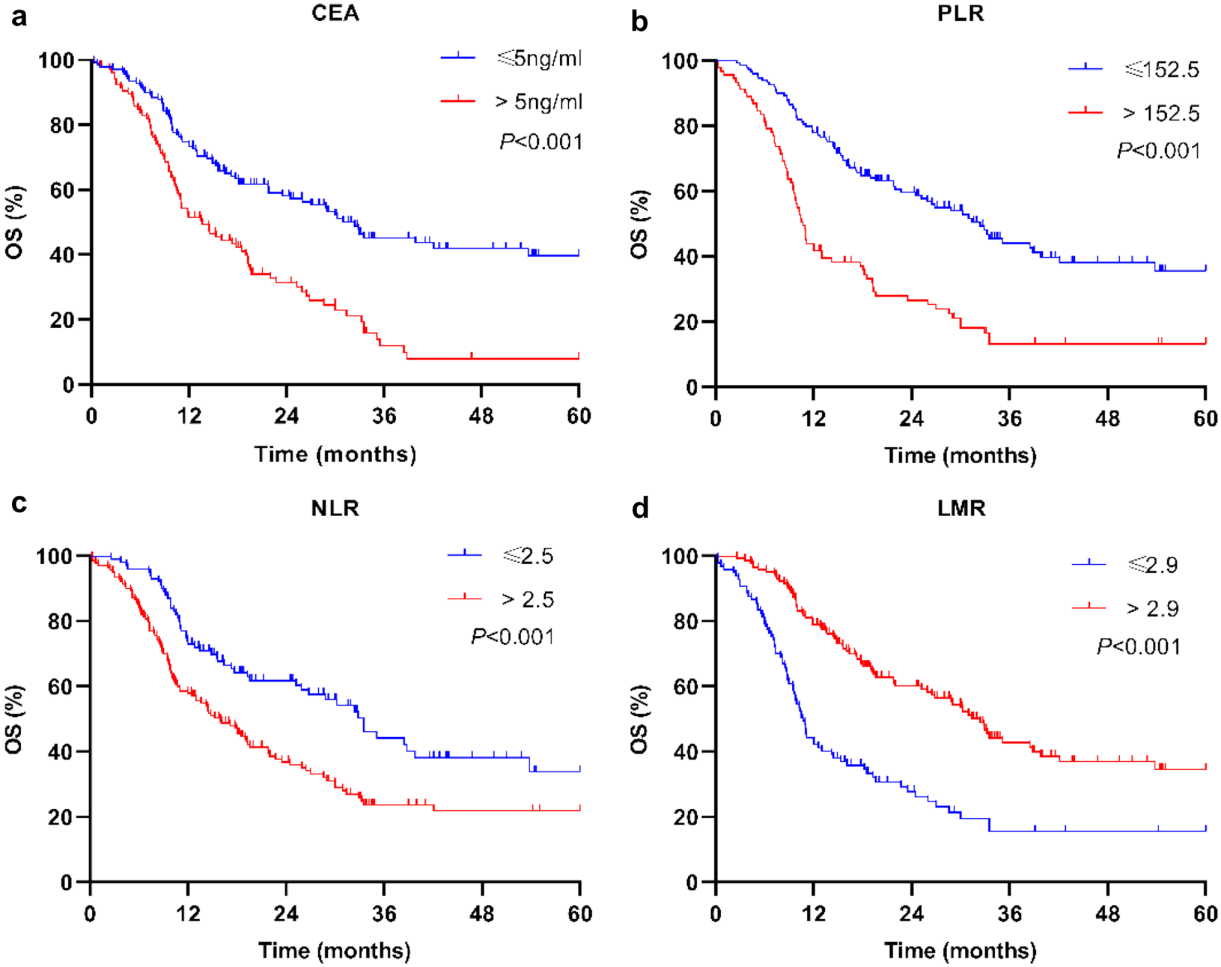
OS for the PNET patients in matched groups using Kaplan–Meier analysis and log-rank test. **a** OS among PNET patients based on low pretreatment CEA (≤ 5 ng/ml) and high pretreatment CEA (> 5 ng/ml). **b** OS among PNET patients based on low pretreatment PLR (≤ 152.5) and high pretreatment PLR (> 152.5). **c** OS among PNET patients based on low pretreatment NLR (≤ 2.5) and high pretreatment NLR (> 2.5). **d** OS among PNET patients based on low pretreatment LMR (≤ 2.9) and high pretreatment LMR (> 2.9). Abbreviations: *OS* overall survival; *PNET* pulmonary neuroendocrine tumor; *CEA* carcinoembryonic antigen; *PLR* platelet–lymphocyte ratio; *NLR* neutrophil–lymphocyte ratio; *LMR* lymphocyte–monocyte ratio

At the end of the follow-up time, PFS was calculated in all 266 patients, but only 248 patients were calculated for OS, and 18 patients were lost to follow-up. A total of 227 (85.34%) patients presented disease progression, and 161 (64.92%) patients died of PNETs. Cox proportional hazards model was used to evaluate the potential predictors, as shown in Table 4. The univariate analysis revealed that PFS was significantly associated with gender (HR 0.553; 95% CI 0.360–0.849; *P* = 0.007), smoking (HR 1.360; 95% CI 1.042–1.774; *P* = 0.024), tumor size (3–5 cm: HR, 1.863; 95% CI 1.349–2.572; *P* = 0.000; 5–7 cm: HR 2.634; 95% CI 1.808–3.838; *P* = 0.000; > 7 cm: HR 2.412; 95% CI 1.545–3.766; *P* = 0.000), metastasis (lymph-node metastasis: HR 4,390; 95% CI 2.788–6.912; *P* = 0.000; distant metastasis: HR 2.824; 95% CI 2.148–3.713; *P* = 0.000), pathotypes (taking SCLC as reference, LCNEC: HR 0.406; 95% CI 0.220–0.749; *P* = 0.004; TC: HR 0.098; 95% CI 0.031–0.310; *P* = 0.000; AC: HR 0.221; 95% CI 0.104–0.472; *P* = 0.000), Ki-67 index (HR 1.016; 95% CI 1.009–1.024; *P* = 0.000), pretreatment CEA (> 5 ng/ml: HR 2.105; 95% CI 1.609–2.754; *P* = 0.000), PLR (> 152.5: HR 1.827; 95% CI 1.391–2.399; *P* = 0.000), NLR (> 2.5: HR 1.562; 95% CI 1.185–2.058; *P* = 0.002), LMR (> 2.9: HR 0.481; 95% CI, 0.366–0.631; *P* = 0.000) and first-line treatment modalities (surgery: HR 0.127; 95% CI 0.064–0.252; *P* = 0.000; surgery combined with chemotherapy or radiotherapy: HR 0.338; 95% CI 0.227–0.506; *P* = 0.000). OS was significantly associated with gender (female: HR 0.488; 95% CI 0.290–0.820; *P* = 0.007), age at diagnosis (≥ 60 years: HR 1.464; 95% CI 1.027–2.088; *P* = 0.035), BMI (18.5–23.9 kg/m^2^: HR, 0.476; 95% CI 0.265–0.856; *P* = 0.013; > 24 kg/m^2^: HR 0.413; 95% CI 0.227–0.752; *P* = 0.004), tumor size (3–5 cm: HR 1.738; 95% CI 1.196–2.526; *P* = 0.004; 5–7 cm: HR, 1.750; 95% CI 1.115–2.747; *P* = 0.015; > 7 cm: HR 1.761; 95% CI 1.041–2.980; *P* = 0.035), metastasis (lymph-node metastasis: HR 5.633; 95% CI 2.963–10.707; *P* = 0.000; distant metastasis: HR 2.761; 95% CI 2.015–3.782; *P* = 0.000), pathotypes (taking SCLC as reference, LCNEC: HR 0.598; 95% CI 0.313–1.144; *P* = 0.120; TC: HR 0.170; 95% CI 0.054–0.538; *P* = 0.003; AC: HR 0.354; 95% CI 0.165–0.759; *P* = 0.008), Ki-67 index (HR 0.014; 95% CI 1.005–1.022; *P* = 0.002), pretreatment CEA (> 5 ng/ml: HR 2.147; 95% CI 1.563–2.947; *P* = 0.000), PLR (> 152.5: HR 2.502; 95% CI 1.820–3.441; *P* = 0.000), NLR (> 2.5: HR 1.798; 95% CI 1.286–2.514; *P* = 0.001), LMR (> 2.9: HR 0.403; 95% CI 0.293–0.555; *P* = 0.000), and first-line treatment modalities (surgery: HR 0.236; 95% CI 0.119–0.468; *P* = 0.000; surgery combined with chemotherapy or radiotherapy: HR, 0.366 95% CI, 0.224–0.597; *P* = 0.000). In multivariate analysis, pretreatment CEA (> 5 ng/ml: HR 1.674; 95% CI 1.141–2.456; *P* = 0.008) and LMR (> 2.9: HR 0.478; 95% CI 0.279–0.820; *P* = 0.007) were identified as independent prognostic factors for PFS. BMI (18.5–23.9 kg/m^2^: HR 0.249; 95% CI 0.088–0.701; *P* = 0.009; > 24 kg/m^2^: HR, 0.262; 95% CI 0.085–0.804; *P* = 0.019), lymph-node metastasis (HR 4.534; 95% CI 1.887–10.894; *P* = 0.001), pathotypes (taking SCLC as reference, LCNEC: HR 3.154; 95% CI 1.182–8.419; *P* = 0.022; TC: HR 0.187; 95% CI 0.036–0.962; *P* = 0.045), pretreatment PLR (> 152.5: HR 2.305; 95% CI 1.311–4.055; *P* = 0.004), and LMR (> 2.9: HR 0.524; 95% CI 0.279–0.985; *P* = 0.045) were identified as independent prognostic factors for OS. The Kaplan–Meier plots were used to generate survival curves (Figs. 2 and 3). The subgroups with pretreatment CEA ≤ 5 ng/ml, PLR ≤ 152.5, NLR ≤ 2.5, and LMR > 2.9 presented a better PFS (Fig. 2) and OS (Fig. 3) (all *P* < 0.05).

**Table 4:** Analysis of potential risk factors for PFS and OS using univariate and multivariate Cox proportional hazard models

Risk Factors	PFS: cox regression analysis (*N* = 266, 227 progression events)				OS: cox regression analysis (*N* = 248, 161 dead events)			
	Univariable analysis		Multivariate analysis		Univariable analysis		Multivariate analysis	
	HR (95% CI)	*P* value	HR (95% CI)	*P* value	HR (95% CI)	*P* value	HR (95% CI)	*P* value
Gender								
Male	Reference	–	Reference	–	Reference	–	Reference	–
Female	0.553(0.360–0.849)	0.007*	1.240 (0.681–2.257)	0.483	0.488 (0.290–0.820)	0.007*	1.073 (0.522–2.202)	0.849
Age at diagnosis (years)								
< 60	Reference	–			Reference	–	Reference	–
≥ 60	1.197 (0.890–1.609)	0.234			1.464 (1.027–2.088)	0.035*	0.583 (0.337–1.010)	0.054
BMI (kg/m^2^)								
< 18.5	Reference	–			Reference	–	Reference	–
18.5–23.9	0.683 (0.393–1.189)	0.177			0.476 (0.265–0.856)	0.013*	0.249(0.088–0.701)	0.009*
> 24	0.554 (0.315–0.974)	0.040*			0.413 (0.227–0.752)	0.004*	0.262(0.085–0.804)	0.019*
Smoking								
No	Reference	–	Reference	–	Reference	–		
Yes	1.360 (1.042–1.774)	0.024*	1.130 (0.770–1.659)	0.531	1.289 (0.939–1.768)	0.116		
Laterality								
Left	Reference	–			Reference	–		
Right	0.991(0.755–1.301)	0.948			0.955 (0.691–1.320)	0.781		
Others	1.817(0.966–3.415)	0.064			1.735 (0.890–3.384)	0.106		
Tumor size (cm)								
≤ 3	Reference	–	Reference	–	Reference	–	Reference	–
3–5	1.863 (1.349–2.572)	0.000*	1.758 (1.079–2.867)	0.024*	1.738 (1.196–2.526)	0.004*	1.686 (0.942–3.016)	0.079
5–7	2.634 (1.808–3.838)	0.000*	1.600 (0.950–2.693)	0.077	1.750 (1.115–2.747)	0.015*	1.362 (0.706–2.627)	0.357
> 7	2.412 (1.545–3.766)	0.000*	1.302 (0.716–2.365)	0.387	1.761 (1.041–2.980)	0.035*	1.099 (0.513–2.354)	0.809
Lymph-node metastasis								
No	Reference	–	Reference	–	Reference	–	Reference	–
Yes	4.390 (2.788–6.912)	0.000*	1.748 (0.939–3.256)	0.078	5.633 (2.963–10.707)	0.000*	4.534 (1.887–10.894)	0.001*
Distant metastasis								
No	Reference	–	Reference	–	Reference	–	Reference	–
Yes	2.824 (2.148–3.713)	0.000*	1.115 (0.751–1.655)	0.590	2.761 (2.015–3.782)	0.000*	1.043 (0.637–1.706)	0.868
Pathotypes								
SCLC	Reference	–	Reference	–	Reference	–	Reference	–
LCNEC	0.406 (0.220–0.749)	0.004*	1.420 (0.632–3.191)	0.396	0.598 (0.313–1.144)	0.120	3.154 (1.182–8.419)	0.022*
TC	0.098 (0.031–0.310)	0.000*	0.123 (0.023–0.664)	0.015*	0.170 (0.054–0.538)	0.003*	0.187 (0.036–0.962)	0.045*
AC	0.221 (0.104–0.472)	0.000*	0.408 (0.141–1.178)	0.097	0.354 (0.165–0.759)	0.008*	0.773 (0.237–2.523)	0.669

Risk Factors	PFS: cox regression analysis (*N* = 266, 227 progression events)				OS: cox regression analysis (*N* = 248, 161 dead events)			
	Univariable analysis		Multivariate analysis		Univariable analysis		Multivariate analysis	
	HR (95% CI)	*P* value	HR (95% CI)	*P* value	HR (95% CI)	*P* value	HR (95% CI)	*P* value
CEA (ng/ml)								
≤ 5	Reference	–	Reference	–	Reference	–	Reference	–
> 5	2.105 (1.609–2.754)	0.000*	1.674 (1.141–2.456)	0.008*	2.147 (1.563–2.947)	0.000*	1.223 (0.782–1.913)	0.378
PLR								
≤ 152.5	Reference	–	Reference	–	Reference	–	Reference	–
> 152.5	1.827 (1.391–2.399)	0.000*	1.374 (0.842–2.244)	0.204	2.502 (1.820–3.441)	0.000*	2.305 (1.311–4.055)	0.004*
NLR								
≤ 2.5	Reference	–	Reference	–	Reference	–	Reference	–
> 2.5	1.562 (1.185–2.058)	0.002*	1.255 (0.812–1.939)	0.307	1.798 (1.286–2.514)	0.001*	1.419 (0.851–2.364)	0.180
LMR								
≤ 2.9	Reference	–	Reference	–	Reference	–	Reference	–
> 2.9	0.481 (0.366–0.631)	0.000*	0.478 (0.279–0.820)	0.007*	0.403 (0.293–0.555)	0.000*	0.524 (0.279–0.985)	0.045*
Treatment strategy								
Chemotherapy	Reference	–	Reference	–	Reference	–	Reference	–
Chemotherapy** + **radiotherapy	0.583 (0.239–1.422)	0.236	1.222 (0.355–4.208)	0.750	0.659 (0.243–1.789)	0.413	1.061 (0.234–4.805)	0.938
Surgery	0.127 (0.064–0.252)	0.000*	0.471 (0.197–1.125)	0.090	0.236 (0.119–0.468)	0.000*	0.865 (0.350–2.136)	0.753
Surgery** + **chemotherapy**/**radiotherapy	0.338 (0.227–0.506)	0.000*	0.437 (0.225–0.849)	0.015*	0.366 (0.224–0.597)	0.000*	0.345 (0.138–0.866)	0.023*
Others	0.808 (0.458–1.425)	0.462	0.746 (0.363–1.537)	0.427	1.140 (0.597–2.177)	0.692	1.094 (0.498–2.402)	0.824
Syn	0.898 (0.572–1.411)	0.641			0.889 (0.535–1.477)	0.651		
CgA	0.883 (0.655–1.190)	0.413			0.824 (0.580–1.170)	0.279		
CK18	2.182 (0.809–5.890)	0.123			2.220 (0.701–7.034)	0.175		
TTF-1	1.126 (0.744–1.704)	0.576			1.065 (0.667–1.700)	0.792		
Napsin A	1.254 (0.515–3.053)	0.618			2.452 (0.996–6.037)	0.051		
CK5/6	1.126 (0.528–2.402)	0.759			1.526 (0.710–3.279)	0.279		
Ki67	1.016 (1.009–1.024)	0.000*	0.996(0.983–1.010)	0.602	1.014 (1.005–1.022)	0.002*	0.997(0.983–1.012)	0.732
P63	1.388 (0.858–2.245)	0.182			1.732 (0.990–3.031)	0.054		

*PFS* progression-free survival; *OS* overall survival; HR, hazard ratio; *95% CI* 95% confidence intervals; *BMI* body mass index; *SCLC* small-cell lung cancer; *LCNEC* large-cell neuroendocrine carcinoma; *AC* atypical carcinoid; *TC* typical carcinoid; *CEA* carcinoembryonic antigen; *PLR* platelet–lymphocyte ratio; *NLR* neutrophil–lymphocyte ratio; *LMR* lymphocyte–monocyte ratio; *Syn* synaptophysin; *CgA* chromogranin A; *CK18* Cytokeratin 18; *TTF-1* thyroid transcription factor-1; *CK5/6* Cytokeratin 5/6

^*^P < 0.05

## Discussion

In our retrospective study, patients presented distinctly different clinical characteristics among the pathotypes of PNETs. SCLC tends to occur among elderly individuals, most frequently with metastasis and have worse prognosis. With regard to immunohistochemical profile, SCLC had a higher positive rate of TTF-1. The Ki-67 index was significantly higher in SCLC and LCNEC than in carcinoid. Moreover, this current research indicated that smoking, pretreatment CEA > 5 ng/ml, and poorly differentiated PNET pathotypes were independent risk factors for lymph-node metastasis. Smoking and pretreatment CEA > 5 ng/ml were independent risk factors for distant metastasis. Survival analysis showed that males, age at diagnosis ≥ 60 years, BMI < 18.5 kg/m^2^, large tumor size, metastasis, poorly differentiated pathotypes, high Ki-67 index, low pretreatment LMR and increase of pretreatment CEA, PLR, and NLR were risk factors for OS. Surgery alone or surgery combined with chemotherapy and/or radiotherapy could prolong survival.

Previous studies have demonstrated the clinicopathological characteristics among subgroups of PNETs. Yeh and Chou (2014) and Kim et al. (2020) confirmed that age at diagnosis, gender, smoking, tumor size, metastasis, stage, and occurring disease progression were significantly different among SCLC, LCNEC, TC, and AC, consistent with our results.

Neuroendocrine marker expression also differed among subtypes of PNETs. TTF-1 was reported to be expressed by surfactant-producing type 2 pneumocytes and can also be detected in most small-cell and approximately 60–80% of lung adenocarcinomas (Bruno et al. 1995; Ordóñez 2000; Yatabe et al. 2002). Furthermore, the Ki-67 index represents the proliferation ability. There is a substantial amount of research, reporting that Ki-67 has great utility for SCLC/LCNEC from carcinoids, but typical and atypical carcinoids cannot be distinguished (Garg et al. 2019; Marchevsky et al. 2018; Naheed et al. 2019). In addition, Thunnissen et al. (2017) suggested that cytokeratin CK8, CK18, CK7, and CK19 may be associated with SCLC. CK5/6, p63, and p40 are commonly used as immunohistochemical markers for squamous cell carcinoma of the lung (Matsukuma et al. 2018). However, there remains conflicting views on the immunohistochemical profile. The 2015 WHO classification of lung tumors suggested that Syn and CgA were recommended as first-hand choice for neuroendocrine markers (Travis et al. 2015), while Staaf et al. (2020) claimed that CgA had limited sensitivity. There are also different views on the prognostic role of immunohistochemical molecules. Hokari et al. (2020) found that highly expressed TTF-1 was associated with poor prognosis. The same results were obtained by Frost et al. (2020) later. In contrast, Dong et al. (2020) indicated that patients with positive expression of Napsin A had longer overall survival time than patients with negative expression, and the other NE markers were not associated with overall survival time. Our data showed a high positive rate of Syn, CgA, CK18, and TTF-1 in PNETs. In addition, the TTF-1 expression and Ki-67 index were significant different among the PNETs subtypes. The positivity of TTF-1 was much higher in SCLC, and the Ki-67 index was significantly higher in SCLC and LCNEC than in carcinoid tumors, which is consistent with the above reports. Besides, the Ki-67 index was proven to be of great significant in predicting PNET prognosis. Nonetheless, we did not find a prognostic role of other immunohistochemical molecules in PNETs. We suspect that the diverse result may be caused by the different study populations or methods used in research. Further research is needed to clarify the immunohistochemical molecular characteristics of PNETs.

Only pretreatment NLR showed significant differences among subgroups of PNETs at baseline, but PLR and LMR showed great prognostic value for PNETs. ROC analysis was performed to identify the optimal cut-off point of PLR, NLR, and LMR, and an optimal PLR, NLR, and LMR cut-off value of 152.5, 2.5, and 2.9, respectively. However, interracial and histological differences in the PLR, NLR, and LMR may influence the cut-off point (Okui et al. 2017). There are different reports on the PLR and NLR cut-off value. Shao and Cai (2015) defined the cut-off value of pretreatment PLR and NLR as 150 and 4.15, respectively. Okui et al. (2017) described the NLR cut-off value as 1.7, Suzuki et al. (2019) and Wang et al. (2020) held different views. The most appropriate cut-off value has not been established, and the current value could be viewed as arbitrary (Okui et al. 2017). Further researches are needed to determine the optimal cut-off value. In addition, pretreatment PLR, NLR, and LMR were first applied to all pathotypes of PNETs for prognostic analysis. Interestingly, we found that low pretreatment LMR, high pretreatment PLR, and NLR were associated with poor PFS and OS. These results were fairly comparable to the results of previous single pathology studies (Okui et al. 2017; Shao and Cai 2015; Suzuki et al. 2019; Wang et al. 2020).

There are several potential hypotheses regarding the mechanism by which low LMR, high NLR, and PLR promote cancer progression. Low LMR, high NLR, and PLR are equivalent to an increase in neutrophil count and a decrease in lymphocytes. Multiple studies have suggested that elevated neutrophil counts or lymphocytes loss may facilitate tumor development and progression by acting on tumor microenvironment. There are two major impacts toward tumors’ progression. On one hand, inflammatory cells themselves play an important role in tumorigenesis and progression. Lymphocytes control many immune functions and lymphocyte loss owing to failed antitumor immunity (Lin and Pollard 2004). A high NLR indicates an imbalance in the immune response, which may impair normal antitumor functions, thus contributing to a worse prognosis for the host (Nagai et al. 2014; Xiao et al. 2013). On the other hand, inflammatory cells play a role in promoting tumor development by secreting a variety of inflammatory cytokines and chemokines, as well as proangiogenic factors (e.g., tumor necrosis factor, interleukin-1, interleukin-6, and vascular endothelial growth factor) (Balkwill and Mantovani 2001; Kusumanto et al. 2003). The exact mechanism of PLR, NLR, and LMR on tumor prognosis remains unclear yet. Further studies still need to be conducted to fully understand the molecular mechanism involved in these results. Inflammation within the tumor microenvironment can affect every aspect of tumor development and progression as well as response to therapy (Greten and Grivennikov 2019). In future research, inflammatory cells may be combined to guide the formulation of immunotherapy or chemotherapy.

For the analysis of the influence factors of survival, except for the above-mentioned immune-inflammation index, this current research showed that pretreatment CEA > 5 ng/ml was associated with metastasis, poor PFS, and OS in PNETs. Multiple studies have reported CEA as a tumor maker in the progression of lung cancer. (Grunnet and Sorensen 2012) pointed out that the serum level of CEA provided prognostic and predictive information on the risk of recurrence and death in NSCLC independent of treatment or study design. Kuo et al. (2020) implied that CEA was a prognostic factor associated with new metastasis, poor PFS, and OS in patients harboring epidermal growth factor receptor (EGFR) mutations. However, a few studies have reported the relationship between CEA and PNETs. Our study further confirmed the prognostic role of CEA. More attention should be given to tumor markers in clinical practice.

In terms of population inclusion, the number of LCNEC and carcinoid cases was relatively small. The immunohistochemical profile was incomplete, and the missing data for neuroendocrine marker expression may weaken the significant difference between groups. In addition, the baseline levels of the involved cases were not completely consistent (such as the age, stage, or first-line treatment modalities), and we did not discuss the guiding role of clinicopathological characteristics in treatment. Further research is needed to explore its guiding role in treatment.

## Conclusions

PNETs are a group of highly heterogeneous tumors with different clinical manifestations, pathological features, and prognoses. Knowing the clinicopathological characteristics and immunophenotypes of PNETs is significant for diagnosis. Pretreatment PLR, LMR, and CEA have certain value in the prognosis of PNETs.

## Data Availability

The datasets generated and analyzed during the current study are available from the corresponding author on reasonable request.

## Abbreviations

PNET: Pulmonary neuroendocrine tumor
SCLC: Small cell lung cancer
LCNEC: Large cell neuroendocrine carcinoma
TC: Typical carcinoid
AC: Atypical carcinoid
PFS: Progression-free survival
OS: Overall survival
CEA: Carcinoembryonic antigen
PLR: Platelet–lymphocyte ratio
NLR: Neutrophil–lymphocyte ratio
LMR: Lymphocyte–monocyte ratio
OR: Odd ratio
HR: Hazard ratio
95% CIs: 95% Confidence intervals
ROC curve: Receiver-operating characteristic curve
AUC: Area under the ROC curve
TTF-1: Thyroid transcription factor-1
Syn: Synaptophysin
CgA: Chromogranin A
CK18: Cytokeratin 18
CK5/6: Cytokeratin 5/6
WHO: World Health Organization
NE: Neuroendocrine
PD-1: Programmed death 1
PD-L1: Programmed death ligand 1
LCs: Lung carcinoids
SEER: Surveillance, Epidemiology, and End Results
NSCLC: Non-small-cell lung cancer
EGFR: Epidermal growth factor receptor
